# Contributions of psychological needs, self-compassion, leisure-time exercise, and achievement goals to academic engagement and exhaustion in Canadian medical students

**DOI:** 10.3352/jeehp.2018.15.2

**Published:** 2018-01-08

**Authors:** Oksana Babenko, Amber Mosewich, Joseph Abraham, Hollis Lai

**Affiliations:** 1 Department of Family Medicine, Faculty of Medicine and Dentistry, University of Alberta, Edmonton, Canada; 2 Faculty of Kinesiology, Sport, and Recreation, University of Alberta, Edmonton, Canada; 3 Department of Dentistry, Faculty of Medicine and Dentistry, University of Alberta, Edmonton, Canada; Hallym University, Korea

**Keywords:** Academic burnout, Self-determination theory, Coping

## Abstract

**Purpose:**

To investigate the contributions of psychological needs (autonomy, competence, and relatedness) and coping strategies (self-compassion, leisure-time exercise, and achievement goals) to engagement and exhaustion in Canadian medical students.

**Methods:**

This was an observational study. Two hundred undergraduate medical students participated in the study: 60.4% were female, 95.4% were 20–29 years old, and 23.0% were in year 1, 30.0% in year 2, 21.0% in year 3, and 26.0% in year 4. Students completed an online survey with measures of engagement and exhaustion from the Oldenburg Burnout Inventory–student version; autonomy, competence, and relatedness from the Basic Psychological Needs Scale; self-compassion from the Self-Compassion Scale–short form; leisure-time exercise from the Godin Leisure-Time Exercise Questionnaire; and mastery approach, mastery avoidance, performance approach, and performance avoidance goals from the Achievement Goals Instrument. Descriptive and inferential analyses were performed.

**Results:**

The need for competence was the strongest predictor of student engagement (β= 0.35, P= 0.000) and exhaustion (β= −0.33, P= 0.000). Students who endorsed mastery approach goals (β= 0.21, P= 0.005) and who were more self-compassionate (β= 0.13, P= 0.050) reported greater engagement with their medical studies. Students who were less self-compassionate (β= −0.32, P= 0.000), who exercised less (β= −0.12, P= 0.044), and who endorsed mastery avoidance goals (β= 0.22, P= 0.003) reported greater exhaustion from their studies. Students’ gender (β= 0.18, P= 0.005) and year in medical school (β= −0.18, P= 0.004) were related to engagement, but not to exhaustion.

**Conclusion:**

Supporting students’ need for competence and raising students’ awareness of self-compassion, leisure-time exercise, and mastery approach goals may help protect students from burnout-related exhaustion and enhance their engagement with their medical school studies.

## Introduction

Medical students are highly motivated to achieve; this is evident in their perseverance with stringent medical school entrance requirements and the educational demands required to become a physician [1]. While some pressures may be external (e.g., a large volume of material to learn), some may come from within (e.g., the use of inadequate coping strategies). As a result, some students can experience academic burnout—feeling emotionally, physically, and cognitively exhausted—and their engagement with their studies becomes suboptimal [2]. Since it may take months for burnout to subside [2], it is likely that its traces will be present following students’ graduation from medical school. Therefore, it is critical to investigate and address the burnout phenomenon in medical school. To do so, we drew on self-determination theory (SDT) [3] to investigate the roles of students’ basic psychological needs for autonomy, competence, and relatedness, and we examined students’ self-compassion [4], leisure-time exercise [5], and achievement goals [6] as emotion-oriented, physical, and cognitive coping strategies, respectively.

According to SDT, 3 basic psychological needs—autonomy, competence, and relatedness—must be supported by the environment to ensure an individual’s optimal functioning in achievement settings [1,3]. Autonomy is the need to experience one’s behaviour as volitional and self-endorsed; competence is the need to experience efficacy and mastery in important activities in one’s life; and relatedness is the need to feel connected with and valued by others [3]. When these needs are supported by the environment, individuals are more likely to initiate and engage effectively in activities that lead to growth and well-being. In contrast, when basic needs are thwarted by the environment, individuals experience low motivation, disengagement, and psychological ill-being [1,3].

Coping strategies are specific efforts that individuals employ in an attempt to manage stressful events. When experiencing negative emotions due to stress or failure, individuals need to find ways to cope with these negative emotions through processes that enhance positive responses and mitigate negative ones [4]. From this point of view, concern for oneself or self-compassion is an emotion-oriented coping strategy conceptualized as treating oneself with kindness and understanding when one has failed or made mistakes (i.e., developing tolerance for imperfection in oneself), seeing one’s own pain and suffering as part of a larger human experience (i.e., recognizing that life is not perfect), and allowing oneself to experience feelings as they are without suppressing or avoiding them [4].

Physical activity is an effective way of coping with stress. When individuals pursue leisure-time exercise, they are much happier and more effectively manage stressors and challenges in life [5,7]. Despite the well-recognized health benefits of physical activity, students’ involvement in leisure-time exercise tends to decline after beginning post-secondary studies [7].

The way individuals respond to and cope with environmental pressures can also be explained by the types of cognitions (implicit goals) individuals develop in achievement settings where a possibility of failure exists [6,8]. Elliot and McGregor [6] identify 4 goals: (1) performance approach (to demonstrate competence relative to others), (2) performance avoidance (to avoid demonstrating incompetence relative to others), (3) mastery approach (to gain new knowledge and skills or to improve on one’s performance), and (4) mastery avoidance (to avoid personal incompetence, with a focus on not doing worse than one has done in the past). As such, achievement goals can be viewed as coping strategies that individuals adopt when they encounter challenges. Mastery approach goals are the most beneficial because they promote interest, deep learning, engagement, and self-regulated learning [6,8]. Performance approach goals, although linked to high achievement, are less adaptive due to their associations with surface learning, cheating, and self-handicapping [6,8]. Avoidance goals are maladaptive, as they relate to low performance, poor engagement, and psychological ill-being [6,8].

In this study, we aimed to answer the following research questions: (1) what are the unique contributions of students’ needs for autonomy, competence, and relatedness to their engagement and exhaustion in medical school? and (2) what are the unique contributions of students’ self-compassion, leisure-time exercise, and achievement goals to their engagement and exhaustion in medical school?

With respect to engagement, we hypothesized that the 3 basic psychological needs (i.e., competence, autonomy, and relatedness), self-compassion, leisure-time exercise, and mastery approach goals would have positive contributions, while performance approach, performance avoidance, and mastery avoidance goals would have negative contributions. With respect to exhaustion, we hypothesized that the 3 basic psychological needs (i.e., competence, autonomy, and relatedness), self-compassion, leisure-time exercise, and mastery approach goals would have negative contributions, while performance approach, performance avoidance, and mastery avoidance goals would have positive contributions.

## Methods

### Ethical approval

This study was approved by the University of Alberta Research Ethics Board (Pro00066510). Participation in the study was voluntary. Informed consent was implied by the overt action of completing the survey after reading the information letter. Students could choose not to respond to a question with no negative consequences to them.

### Study design

This was an observational survey study.

### Participants

Two hundred undergraduate medical students (Table 1) at the University of Alberta, Canada, completed an online survey.

### Measures

Publicly available, non-proprietary instruments were used in this study. The survey items, adapted as described below for the purposes of this study, are shown in Table 2. The 16-item Oldenburg Burnout Inventory–student version [2] was used to measure engagement (8 items) and exhaustion (8 items). Using a 4-point Likert-type scale (1, strongly disagree; 4, strongly agree), students were asked to indicate their level of agreement with each statement in relation to their medical program. Higher scores were indicative of greater engagement (α= 0.78) and exhaustion (α= 0.82), respectively.

The 12-item Basic Psychological Needs Scale [9] was used to assess the levels of satisfaction of 3 psychological needs. Each need—autonomy (α=0.75), competence (α=0.79), and relatedness (α=0.89) —was measured by 4 items. Students were asked to indicate how they typically felt in relation to their medical program, using a 6-point Likert-type scale (1, strongly disagree; 6, strongly agree). Higher scores indicated greater satisfaction of the respective needs.

The 12-item Self-Compassion Scale–short form [10] was used to measure the degree of compassion students exhibited toward themselves in instances of failure or during a challenging time. Using a 5-point Likert-type scale (1, almost never; 5, almost always), students were asked to indicate how often they behaved in a certain way. Higher scores were indicative of greater self-compassion (α= 0.86).

The Godin Leisure-Time Exercise Questionnaire [5] was used to measure students’ leisure-time exercise habits. Using one of the response options (0, none; 1, 1-3 times a week; 2, 4-6 times a week; 3, 7 times a week or more), students were asked to indicate the number of times they engaged in mild, moderate, and strenuous leisure-time exercise bouts of at least 15 minutes of duration in a typical week; examples of such activities were provided for each intensity category. The number of bouts at each intensity level was then multiplied by 3, 5, and 9 metabolic equivalents (for mild, moderate, and strenuous activity, respectively) and summed to derive a leisure-time exercise score for each student. Higher scores were indicative of greater involvement (frequency and/or intensity) in leisure-time exercise.

The Achievement Goals Instrument [11] was used to measure students’ mastery approach, mastery avoidance, performance approach, and performance avoidance goals (19 items in total). This measure was chosen because it assessed students’ achievement goals in relation to their medical program as opposed to their goals in individual courses. Minor changes were made in the item wording to better reflect the nature of medical education (e.g., ‘coworkers,’ ‘projects,’ and ‘job’ were replaced with ‘others in my program,’ ‘tasks,’ and ‘program,’ respectively). Using a 7-point Likert-type scale (1, not at all true of me; 7, yes, very true of me), students were asked to indicate the extent to which each statement was true of them in relation to their medical program. Higher scores indicated greater endorsement of the respective achievement goals (α-values, 0.68 to 0.83).

### Analyses

Descriptive analyses were performed for each variable (Tables 1 and 3). Hierarchical regression analyses consisting of 2 successive steps were conducted to examine the unique contributions of individual variables to student engagement and exhaustion. In the first step, the 3 psychological needs were entered to test their contributions to engagement and exhaustion, while accounting for student gender (0, male; 1, female) and year in the medical program. In the second step, self-compassion, leisure-time exercise, and the 4 achievement goals were entered to test their contributions to engagement and exhaustion (while controlling for the variables entered in the first step). An α level of 0.05 was used to determine statistical significance. Analyses were performed using IBM SPSS ver. 24.0 (IBM Corp., Armonk, NY, USA).

## Results

As shown in Table 3, the students who participated in this study had on average a high level of engagement and a moderate level of exhaustion. The correlation between engagement and exhaustion was negative and significant (r= −0.56, P= 0.000). Moderately strong correlations (r> 0.30) were observed between engagement and autonomy, competence, self-compassion, and mastery approach goals. With respect to exhaustion, moderately strong correlations were observed between exhaustion and autonomy, competence, leisure-time exercise, and mastery avoidance goals (Table 3).

The results of the regression analyses of engagement and exhaustion are shown in Tables 4 and 5, respectively. Of the 3 psychological needs, competence had the largest contribution to explaining engagement (β= 0.35, P= 0.000). Students’ gender, year in school, and autonomy were found to be significant in explaining engagement; however, their individual contributions were small (Table 4). When self-compassion, leisure-time exercise, and the 4 achievement goals were entered, mastery approach goals (β= 0.21, P= 0.005) and self-compassion (β= 0.13, P= 0.050) were positively related to engagement.

With respect to exhaustion (Table 5), of the 3 psychological needs, competence made the largest contribution (β= −0.33, P= 0.000), followed by autonomy (β= −0.17, P= 0.010). When self-compassion, leisure-time exercise, and the 4 achievement goals were entered, self-compassion (β=−0.32, P=0.000), leisure-time exercise (β=−0.12, P= 0.044), and mastery avoidance goals (β= 0.22, P= 0.003) were found to be significant in explaining exhaustion. Students’ gender and year in medical school did not make a significant contribution to explaining exhaustion. The raw data are available in Supplement 1.

## Discussion

We highlight 3 findings. First, the need for competence appeared to be the strongest explanatory factor for student engagement and exhaustion. The published literature indicates that setting an optimal level of challenge and providing meaningful, constructive, and timely feedback help fulfill students’ need for competence [1]. This, in turn, is likely to foster students’ engagement with their studies, and could protect students from emotional, physical, and cognitive exhaustion. Of note is the positive relationship between relatedness and exhaustion, which may, in fact, speak to the contagious nature of burnout. It is possible that one may feel related to others through feeling burned out.

Second, those students who endorsed mastery approach goals and who were more self-compassionate reported greater engagement with their studies. In contrast, students who were less self-compassionate, who exercised less, and who endorsed mastery avoidance goals reported greater exhaustion from their studies.

Third, students’ gender and year in the medical program were found to be related to engagement, but not to exhaustion. The finding that gender was not related to exhaustion is consistent with previously published literature examining burnout in medical students and residents [12]. Interestingly, female students in this study exhibited greater engagement with their studies than male students, suggesting that male students may be more prone to becoming disengaged from their studies than female students. The reasons for this have yet to be determined and require future research efforts.

The published literature, although sparse due to the lack of long-term longitudinal studies, suggests that burnout follows a developmental process that may be initiated during students’ academic studies [2]. However, cross-sectional studies have reported no significant differences in the levels of burnout between students and residents [12]. In this study, students appeared to have comparable levels of exhaustion across the 4 years of school; however, the results of the regression analyses showed that students’ engagement with their studies tended to decrease, which may speak to the developmental nature of burnout and/or changes in students’ priorities from studies to job opportunities/residency positions.

Taken together, these findings have important implications for practice in medical education. Specifically, teaching medical students how to effectively and healthily manage their own feelings of fear, anger, shame, inadequacy, and failure is necessary to help protect students from experiencing academic burnout as they undergo intense professional training. Next, to create learning environments that are supportive of students’ need for competence, educators should explicitly discuss the types of goals and mindsets that they wish to instill in their learners (i.e., mastery approach) by reframing failures and mistakes as opportunities for learning and emphasizing effort as the path to mastery. Further, medical programs need to be aware that students who experience exhaustion are also likely to endorse mastery avoidance goals, which are not supportive of the lifelong learning mandate of the medical profession [13]. Finally, in light of the existing evidence that students’ leisure-time exercise habits deteriorate upon entry into college [7], an important question arises as to what curricular initiatives and learning environments are effective in helping students establish and maintain beneficial exercise habits, and by extension, enhance their well-being.

With respect to limitations, this study employed self-reported data, which can introduce bias in students’ responses. Due to the correlational nature of the data and the self-perpetuating process of burnout, causality cannot be inferred from the observed relationships. Nevertheless, it is important that we strive to understand the underpinnings of student engagement and exhaustion so that effective interventions can be developed. Our findings suggest that creating learning environments that are supportive of students’ need for competence and raising students’ awareness of the roles of self-compassion, leisure-time exercise, and mastery approach goals are likely to protect students from exhaustion and to enhance their engagement with their studies.

## Figures and Tables

**Table 1.** Participant characteristics

Characteristic	%
Gender (n = 192)	
Female	60.4
Male	39.6
Age (yr) (n = 194)	
20–24	63.4
25–29	32.0
30–34	4.1
35–39	0.5
Year in program (n = 200)	
Year 1	23.0
Year 2	30.0
Year 3	21.0
Year 4	26.0

**Table 2.** Survey items

Variable	Items	
Academic burnout		
Engagement	1. I find my studies to be a positive challenge.	
	2. This is the only field of study that I can imagine myself doing.	
	3. I always find new and interesting aspects in my studies.	
	4. I feel more and more engaged in my studies.	
	5. It happens more and more often that I think about my studies in a negative way.^a)^	
	6. Sometimes I feel sickened by my studies.^a)^	
	7. Over time, I can see myself becoming disconnected from this type of studies.^a)^	
	8. Lately, I tend to think less about my school tasks and do them almost mechanically.^a)^	
Exhaustion	1. I can tolerate the pressure of my classes very well.^a)^	
	2. When I engage in school work, I usually feel energized.^a)^	
	3. When I am studying or doing school work, I often feel emotionally drained.	
	4. Usually I can manage the amount of my school work very well.^a)^	
	5. After class/school work, I usually feel worn out and weary.	
	6. After my class/school work, I have enough energy for my leisure activities.^a)^	
	7. There are days when I feel tired before arriving at school.	
	8. After class/school work, I tend to need more time than in the past to relax and feel better.	
Basic psychological needs		
Autonomy	1. In my program, I feel free to make decisions.	
	2. In my program, I can use my judgment when solving problems.	
	3. In my program, I can take on responsibilities.	
	4. In my program, I feel free to execute tasks in my own way.	
Competence	1. In my program, I have the ability to do my work well.	
	2. In my program, I feel competent.	
	3. In my program, I am able to solve problems.	
	4. I succeed in my program.	
Relatedness	1. When I am with the people from my program, I feel understood.	
	2. When I am with the people from my program, I feel heard.	
	3. When I am with the people from my program, I feel as though I can trust them.	
	4. When I am with the people from my program, I feel I am a friend to them.	
Self-compassion	1. When I fail at something important to me, I become consumed by feelings of inadequacy.^a)^s	
	2. I try to be understanding and patient toward those aspects of my personality that I do not like.	
	3. When something painful happens, I try to take a balanced view of the situation.	
	4. When I am feeling down, I tend to feel like most other people are probably happier than I am.^a)^	
	5. I try to see my failings as part of the human condition.	
	6. When I am going through a very hard time, I give myself the caring and tenderness I need.	
	7. When something upsets me, I try to keep my emotions in balance.	
	8. When I fail at something that is important to me, I tend to feel alone in my failure.^a)^	
	9. When I am feeling down, I tend to obsess and fixate on everything that is wrong.^a)^	
	10. When I feel inadequate in some way, I try to remind myself that feelings of inadequacy are shared by most people.	
	11. I am disapproving and judgmental about my flaws and inadequacies.^a)^	
	12. I am intolerant and impatient towards those aspects of my personality that I do not like.^a)^	
Leisure-time exercise	In a typical week, how many times do you do the following kinds of exercise for more than 15 minutes during your free time?	
		1. Mild exercise (minimal effort; e.g., easy walking, yoga, golf, snow-mobiling, etc.)
		2. Moderate exercise (not exhausting; e.g., fast walking, easy bicycling, easy swimming, dancing, badminton, etc.)
		3. Strenuous exercise (heart beats rapidly; e.g., running, hockey, basketball, cross-country skiing, long-distance bicycling, vigorous swimming, heavy weights lifting, etc.)
Achievement goals		
Performance approach	1. I prefer to work on tasks where I can show my competence to others.	
	2. I enjoy when others in my program are aware of how well I am doing.	
	3. I like to show that I can perform better than others in my program.	
	4. I try to figure out what it takes to prove my ability to others in my program.	
Performance avoidance	1. I prefer to avoid situations in my program where I might perform poorly.	
	2. I am concerned about taking on a task if my performance would reveal that I had low ability.	
	3. Avoiding a show of low ability is more important to me than learning a new skill.	
	4. I would avoid taking on a new task if there was a chance that I would appear incompetent to others.	
Mastery approach	1. When given a choice, I am willing to select challenging assignments from which I can learn a lot.	
	2. I am willing to step out of my comfort zone if it will help me develop my competence.	
	3. I often look for opportunities to develop new skills and knowledge.	
	4. I enjoy difficult tasks in my program where I will learn new skills.	
Mastery avoidance	1. In my program, I focus on not doing worse than I have done in the past.	
	2. I just hope I am able to master just enough skills so I am competent in my work.	
	3. In my program, I often feel that I am unable to master what is necessary to do my work.	
	4. I avoid taking on new tasks when I am not sure I will be able to master them.	
	5. I often find myself focused on avoiding making mistakes.	
	6. In my program, I often think that I have missed learning the skills necessary to do my work.	
	7. When I am engaged in a task at school, I often think about what I need to do to not mess up.	

a) indicates reverse-coded items.

**Table 3.** Descriptive statistics (means, SDs, correlations, and internal consistency values [α]) for the variables analyzed in this study (n=200)

Variable	Mean±SD	Autonomy	Competence	Relatedness	Self-compassion	Leisure-time exercise	PAP goals	PAV goals	MAP goals	MAV goals	Engagement	Exhaustion
Autonomy	4.35 ± 0.73	*0.75*										
Competence	4.61 ± 0.62	0.46^**^	*0.79*									
Relatedness	4.65 ± 0.87	0.33^**^	0.26^**^	*0.89*								
Self-compassion	3.15 ± 0.64	0.24^**^	0.24^**^	0.30^**^	*0.86*							
Leisure-time exercise	16.2 ± 9.06	0.01	0.00	–0.01	0.08	-						
PAP goals	4.07 ± 1.13	0.05	0.09	–0.03	–0.19^**^	–0.02	*0.73*					
PAV goals	3.42 ± 1.11	–0.12	–0.22^**^	–0.17^*^	–0.23^**^	–0.16^*^	0.30^**^	*0.83*				
MAP goals	5.50 ± 0.75	0.15^*^	0.32^**^	0.14	0.13	0.16^*^	0.05	–0.48^**^	*0.72*			
MAV goals	4.23 ± 0.85	–0.16^*^	–0.43^**^	–0.17^*^	–0.31^**^	–0.16^*^	0.26^*^	0.60^**^	–0.35^**^	*0.68*		
Engagement	2.98 ± 0.46	0.35^**^	0.49^**^	0.20^**^	0.32^**^	0.24^**^	–0.03	–0.19^**^	0.32^**^	–0.25^**^	*0.78*	
Exhaustion	2.58 ± 0.46	–0.41^**^	–0.54^**^	–0.13	–0.25^**^	–0.44^**^	–0.09	0.16^**^	–0.25^**^	0.40^**^	–0.56^**^	*0.82*

Internal consistency values (α) are shown in italics along the main diagonal of the correlation matrix, except for the composite measure of leisure-time exercise. SD, standard deviation; PAP, performance approach; PAV, performance avoidance; MAP, mastery approach; MAV, mastery avoidance.

* P<0.05.

** P<0.01.

**Table 4.** Results of regression analyses: student engagement

Variable	Step 1			Step 2		
	B	SE(B)	β	B	SE(B)	β
Gender	0.17	0.06	0.18^**^	0.17	0.06	0.18^**^
Year in program	–0.06	0.03	–0.14^*^	–0.08	0.03	–0.18^**^
Autonomy	0.10	0.05	0.15^*^	0.09	0.05	0.14^*^
Competence	0.30	0.05	0.41^**^	0.26	0.06	0.35^**^
Relatedness	0.02	0.03	0.04	–0.00	0.03	–0.00
Self-compassion				0.09	0.05	0.13^*^
Leisure-time exercise				–0.01	0.01	–0.11
Performance approach goals				–0.01	0.03	–0.03
Performance avoidance goals				–0.00	0.04	0.00
Mastery approach goals				0.13	0.05	0.21^**^
Mastery avoidance goals				0.03	0.05	0.05
	F (5,186)=15.91, R^2^ adj=0.28, P<0.001			F (11,180)=9.00, R^2^ adj=0.32, P<0.001		

Step 1 includes students’ gender, year in medical school, and the 3 psychological needs; step 2 includes self-compassion, leisure-time exercise, and the 4 achievement goals, while controlling for the variables entered in step 1.

* P<0.05.

** P<0.01.

**Table 5.** Results of regression analyses: student exhaustion

Variable	Step 1			Step 2		
	B	SE(B)	β	B	SE(B)	β
Gender	–0.00	0.06	–0.00	–0.02	0.05	–0.02
Year in program	–0.00	0.03	–0.00	0.00	0.02	0.00
Autonomy	–0.11	0.05	–0.17^*^	–0.11	0.04	–0.17^*^
Competence	–0.35	0.05	–0.49^**^	–0.24	0.05	–0.33^**^
Relatedness	0.04	0.03	0.09	0.08	0.03	0.15^*^
Self-compassion				–0.23	0.04	–0.32^**^
Leisure-time exercise				–0.01	0.01	–0.12^*^
Performance approach goals				–0.04	0.02	–0.10
Performance avoidance goals				–0.06	0.03	–0.11
Mastery approach goals				–0.04	0.04	–0.06
Mastery avoidance goals				0.12	0.04	0.22^**^
	F (5,186)=18.11, R^2^ adj=0.31, P<0.001			F (11,180)=15.04, R^2^ adj=0.45, P<0.001		

Step 1 includes students’ gender, year in medical school, and the 3 psychological needs; step 2 includes self-compassion, leisure-time exercise, and the 4 achievement goals, while controlling for the variables entered in step 1.

* P<0.05.

** P<0.01.

## Supplementary material

Supplement 1. Data files are available from https://doi.org/10.7910/DVN/JHZDLR

Supplement 2. Audio recording of the abstract.

## References

[1] Ten Cate TJ, Kusurkar RA, Williams GC (2011). How self-determination theory can assist our understanding of the teaching and learning processes in medical education: AMEE guide No. 59. Med Teach.

[2] Reis D, Xanthopoulou D, Tsaousis I (2015). Measuring job and academic burnout with the Oldenburg Burnout Inventory (OLBI): factorial invariance across samples and countries. Burn Res.

[3] Ryan RM, Deci EL (2017). Self-determination theory: basic psychological needs in motivation, development, and wellness.

[4] Neff KD, Leary MR, Hoyle RH (2009). Self-compassion. Handbook of individual differences in social behavior.

[5] Shephard R (1997). Godin leisure-time exercise questionnaire. Med Sci Sports Exerc.

[6] Elliot AJ, McGregor HA (2001). A 2 X 2 achievement goal framework. J Pers Soc Psychol.

[7] Decamps G, Boujut E, Brisset C (2012). French college students’ sports practice and its relations with stress, coping strategies and academic success. Front Psychol.

[8] Elliot AJ, Hulleman CS, Elliot AJ, Dweck CS, Yeager DS (2017). Achievement goals. Handbook of competence and motivation: theory and application.

[9] Brien M, Forest J, Mageau GA, Boudrias JS, Desrumaux P, Brunet L, Morin EM (2012). The basic psychological needs at work scale: measurement invariance between Canada and France. Appl Psychol Health Well Being.

[10] Raes F, Pommier E, Neff KD, Van Gucht D (2011). Construction and factorial validation of a short form of the self-compassion scale. Clin Psychol Psychother.

[11] Baranik LE, Barron KE, Finney SJ (2007). Measuring goal orientation in a work domain: construct validity evidence for the 2×2 framework. Educ Psychol Measur.

[12] Richardson DA, Jaber S, Chan S, Jesse MT, Kaur H, Sangha R (2016). Self-compassion and empathy: impact on burnout and secondary traumatic stress in medical training. Open J Epidemiol.

[13] Babenko O, Szafran O, Koppula S, Au L (2017). Motivations for learning of family medicine residents trained in competency-based education. Educ Prim Care.

